# A comparative study of zero-shot inference with large language models and supervised modeling in breast cancer pathology classification

**DOI:** 10.21203/rs.3.rs-3914899/v1

**Published:** 2024-02-06

**Authors:** Madhumita Sushil, Travis Zack, Divneet Mandair, Zhiwei Zheng, Ahmed Wali, Yan-Ning Yu, Yuwei Quan, Atul J. Butte

**Affiliations:** 1 Bakar Computational Health Sciences Institute, University of California, San Francisco, USA; 2 Helen Diller Family Comprehensive Cancer Center, University of California, San Francisco, USA; 3 University of California, Berkeley, USA; 4 Center for Data-driven Insights and Innovation, University of California, Office of the President, Oakland, CA, USA; 5 Department of Pediatrics, University of California, San Francisco, CA, USA

## Abstract

Although supervised machine learning is popular for information extraction from clinical notes, creating large, annotated datasets requires extensive domain expertise and is time-consuming. Meanwhile, large language models (LLMs) have demonstrated promising transfer learning capability. In this study, we explored whether recent LLMs can reduce the need for large-scale data annotations. We curated a manually labeled dataset of 769 breast cancer pathology reports, labeled with 13 categories, to compare zero-shot classification capability of the GPT-4 model and the GPT-3.5 model with supervised classification performance of three model architectures: random forests classifier, long short-term memory networks with attention (LSTM-Att), and the UCSF-BERT model. Across all 13 tasks, the GPT-4 model performed either significantly better than or as well as the best supervised model, the LSTM-Att model (average macro F1 score of 0.83 vs. 0.75). On tasks with a high imbalance between labels, the differences were more prominent. Frequent sources of GPT-4 errors included inferences from multiple samples and complex task design. On complex tasks where large annotated datasets cannot be easily collected, LLMs can reduce the burden of large-scale data labeling. However, if the use of LLMs is prohibitive, the use of simpler supervised models with large annotated datasets can provide comparable results. LLMs demonstrated the potential to speed up the execution of clinical NLP studies by reducing the need for curating large annotated datasets. This may increase the utilization of NLP-based variables and outcomes in observational clinical studies.

## Introduction

Over the last decade, supervised machine learning methods have been the most popular technique for information extraction from clinical notes^1^. However, supervised learning for clinical text is arduous, requiring curation of large domain-specific datasets, interdisciplinary collaborations to design and execute standardized annotation schema, and significant time from multiple domain experts for the meticulous task of data annotation. Supervised modeling can often require subsequent iterative development driven by advanced technical expertise, which can be limiting for certain practitioners. The entire process thus takes a significant amount of time between problem conception and obtaining final results. These challenges, combined with the limited availability of clinical notes corpora, have contributed to an under-utilization of Natural Language Processing (NLP) in observational studies from Electronic Health Records (EHRs)^2^.

Recently, language models have demonstrated promising transfer learning ability, which is encouraging for information extraction from text without extensive task-specific model training^3–5^. Prompt-based inference is popular with generative language models, where practitioners can simply query the model in natural language to obtain the desired information, sometimes by presenting a few examples of the task they may be trying to solve. Prompt-based inference with large language models (LLMs) like the GPT-4 model have demonstrated varying levels of proficiency in medical inference tasks, such as diagnosing complex clinical cases^6–8^, radiology report interpretation^9,10^, clinical notes-based patient phenotyping^11–13^, automated clinical trial matching^14,15^, and improving patient interaction with health systems^16^. However, to understand whether LLMs may be able to alleviate the need to curate large training datasets, few studies have investigated whether zero-shot modeling with LLMs can perform as well as supervised learning in low-resource settings. In this study, utilizing a large corpus of breast cancer pathology notes, we investigate whether large language models can alleviate the need to curate large training datasets for supervised learning to extract information from pathology text. To this end, we have a three-fold contribution:

1. We developed an annotation schema and detailed guidelines to create an expert-annotated dataset of 769 breast cancer pathology reports with document-level, treatment-relevant information. We further analyzed the curation process to identify frequent modes of disagreements in data annotation, which we additionally present here.
2. To establish a baseline of automated breast cancer pathology classification against that of expert clinicians, using the newly curated dataset, we benchmarked the performance of supervised machine learning models of varied levels of complexity, which include a random forest classifier, a long short-term memory network (LSTM) classifier, and a transformers-based medically-trained BERT classifier.
3. We finally prompted the GPT-4 and GPT-3.5-turbo models to obtain *zero-shot* classification results, i.e., results without using any domain-specific manually labeled dataset, which we compared to the supervised learning performance obtained earlier.

## Materials and Methods

### Data

**Figure 1:**
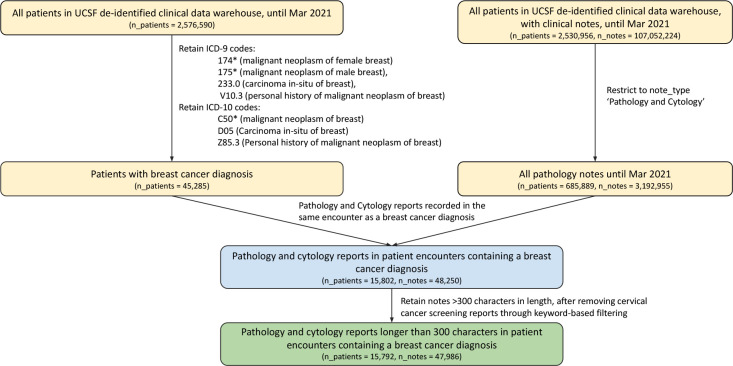
Flow diagram representing inclusion and exclusion criteria for breast cancer pathology report selection before data annotation. Number of patients and number of clinical notes is represented at each stage. The final annotated subset represents a random sample of the final representative dataset obtained in this manner.

Breast cancer pathology reports between 2012 and March 2021 were retrieved from the University of California, San Francisco (UCSF) clinical data warehouse, deidentified and date-shifted with the Philter algorithm as previously described ^17^. Patients with breast cancer were identified by querying for encounters with the ICD-9 codes 174, 175, 233.0, or V10.3, or the ICD-10 codes C50, D05, or Z85.3. The cohort was restricted to pathology reports by selecting the note type ‘Pathology and Cytology’. Notes shorter than 300 characters in length, and those unrelated to breast cancer, for example, those about regular cervical cancer screening through pap smears, were removed through keyword search. A flow diagram for the inclusion and exclusion criteria is presented in Figure 1. Among the final set of notes, 769 pathology reports were randomly selected for manual labeling with treatment-relevant breast cancer pathology.

**Figure 2:**
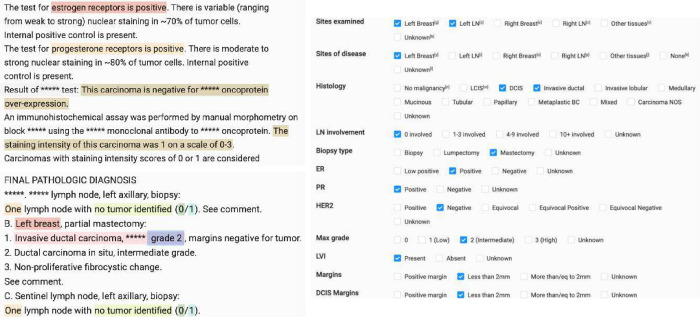
Sample of an annotated pathology report, along with the corresponding document-level annotation schema. *Irrelevant note* refers to those that are not related to a breast cancer diagnosis (further details in the annotation guidelines). The *Unknown* labels refer to the cases where a label could not be inferred based on the information provided in the pathology report.

Annotation schema and guidelines were designed in collaboration with oncology experts to label treatment-relevant breast cancer pathology details. To align with the clinical decision-making process, focus was established on identifying the most aggressive, or “worst” specimen before making document-level inferences. To analyze categories relevant for prognostic inference, categories such as final tumor margins and lymphovascular invasion were added in addition to commonly investigated categories of biomarkers, histopathology, and grade. The final cohort of 769 breast cancer pathology reports was annotated through thirteen key tasks, including ten single-label tasks and three multi-label tasks (Figure 2). Each report mentioned metadata such as the report date and patient ID, along with the pathologist’s comments and the complete clinical diagnosis. Text spans corresponding to cancer stage, lymph node involvement, and tumor-related information were pre-highlighted within text with an external long-short term networks model that had been previously trained for named entity recognition. To establish a good inter-annotator agreement, a group of 2 independent oncology fellows annotated the documents jointly in the first phase. After achieving high inter-annotator agreement, the fellows further labeled the documents in the training subset (570 documents) independently. Furthermore, the test subset (100 reports) was established with documents that were annotated by both oncology fellows, and any disagreements between discordant labels were manually adjudicated by a third reviewer. Similarly, the validation subset (99 reports) was annotated in parallel by 3 medical students and any disagreements were independently adjudicated by the same reviewer. The complete annotation guidelines are provided in the Supplementary Materials (Section S1).

### Zero-shot inference with LLMs

Two large language models, the GPT-3.5 model and the GPT-4 model^18^, were queried via the HIPAA-compliant Azure OpenAI Studio^1^ to provide the requested category of breast cancer pathology information from a given pathology report. No data was permanently transferred to or stored by either OpenAI or Microsoft for any purposes, as previously described^2^. Model inputs were provided in the format *{system role description} {note section text, prompt}*. The specific prompt, model version, and the model hyperparameters are provided in the Supplementary Materials, section S2. All thirteen classification labels were requested through a single prompt, as one call to the model for each pathology report. Prompt development was performed on the development set, and the final results were reported on a held-out test set. Model outputs were requested in the JSON format, which were post-processed into python dictionaries to automatically evaluate model outputs.

### Supervised Modeling

Supervised machine learning classifiers were trained independently for each of the 13 breast cancer pathology classification tasks. Three models of varied complexity were included in the analysis — a random forests classifier^19^, a Long Short Term Memory networks (LSTM) classifier with attention^20,21^, and a fine-tuned UCSF-BERT (base) model^22,23^. The random forests model was initialized with a TF-IDF vector of n-grams within pathology notes, and the word embeddings in the LSTM model were initialized with fasttext^24^ embeddings of 250 dimensions, trained on a corpus of 110 million clinical notes at UCSF. The UCSF-BERT model was pretrained from scratch on 75 million clinical notes at UCSF, and fine-tuned further on pathology classification-specific tasks. Pathology reports were pre-processed to remove punctuations and symbols, and were converted to lowercase before vectorization for the random forests and the LSTM models. For random forests single-label tasks, training data samples of the minority classes were up-sampled to reflect a uniform distribution and address data imbalance. Validation and test data were not modified and reflected the real-world distribution. To find the best parameters for the random forest model, a random grid search was performed, using 3-fold cross-validation on the training data and 15 iterations. For deep learning classifiers, the validation subset was used for hyperparameter tuning. To address the data imbalance in multi-label tasks, asymmetric loss^25^ was used by the deep learning models during model training, and the categorical cross-entropy loss was used for single-label tasks. Further details on model settings and hyperparameter tuning are available in the Supplementary Materials, section S3. Source code for model implementation is further available through the github repository: https://github.com/MadhumitaSushil/BreastCaPathClassification. To obtain a reliable estimate of minority class performance, model performance was evaluated on a held-out test set with the metric macro-averaged F1-score instead of accuracy or micro-averaged F1 score.

## Results

### Breast cancer pathology information extraction dataset

769 breast cancer pathology reports were annotated with detailed breast cancer pathology information across 13 key tasks (Figure 2). Minimum, maximum, mean and median document length of the dataset were 36, 4430, 723.4, and 560 words respectively, and the inter-quartile range was 508 words. The dataset included a diverse population across demographics and age, with nearly 1% of cases being male breast cancer, which reflects the relative incidence of this disease (Table 1). Median patient age was 55 years. To encourage reproducibility and further research, upon manuscript publication, the dataset will be freely shared through the controlled-access repository PhysioNet. Average inter-annotator agreement, as quantified with Krippendorf’s alpha^3^^26^, was 0.85 (Table 2), with variability across tasks. Classification of *DCIS margins* and the multi-label category of *sites examined* showed the most interannotator discordance, while *lympho-vascular invasion* and *invasive carcinoma margin status* showed the highest concordance.

Sources of disagreements between annotators in the deelopment and the test sets were analyzed by an independent adjudicator. Common sources of disagreements included differences in inferring the most aggressive (“worst”) sample when multiple samples were analyzed, incorrectly including information from patient history for providing labels for the current report, linguistic or clinical ambiguity in the pathology report, discordant interpretation of procedures involving excisions when differentiating between a histopathology report and a cytology report, inconsistencies in categorizing metastatic disease sites as “other tissues” and histology as “others”, and inconsistent execution of the annotation guidelines for annotating molecular pathology reports and grade information.

**Figure 3:**
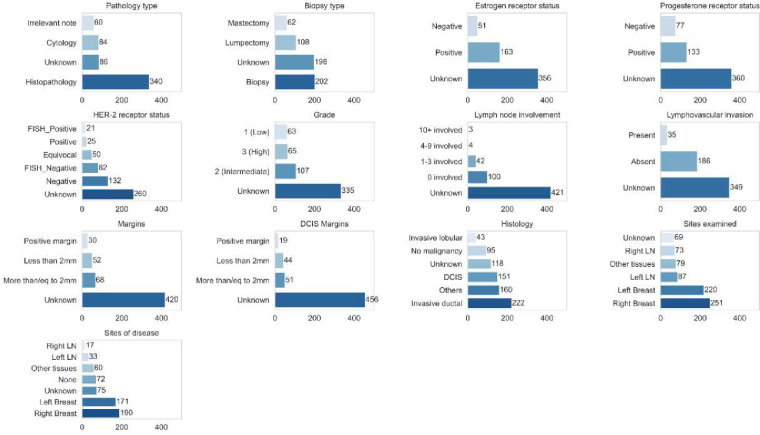
Class distribution for all tasks in the training data for supervised classification.

The class distribution across the annotated data was highly skewed, resulting in a highly imbalanced dataset. Certain low frequency categories, such as groups of histology codes or the *low positive* and *positive* categories of Estrogen Receptor status were combined before further automated classification, resulting in the final distribution presented in Figure 3. The *Unknown* class, which corresponded to the case where the requested information could not be inferred from the given note, was the majority class across 8 of 13 tasks. Among the remaining classes, high imbalances were observed in tasks of inferring the category of the number of lymph nodes involved, lymphovascular invasion, tumor margins, and HER-2 receptor status.

### The GPT-4 model is as good as or better than supervised models in breast cancer pathology classification

**Figure 4:**
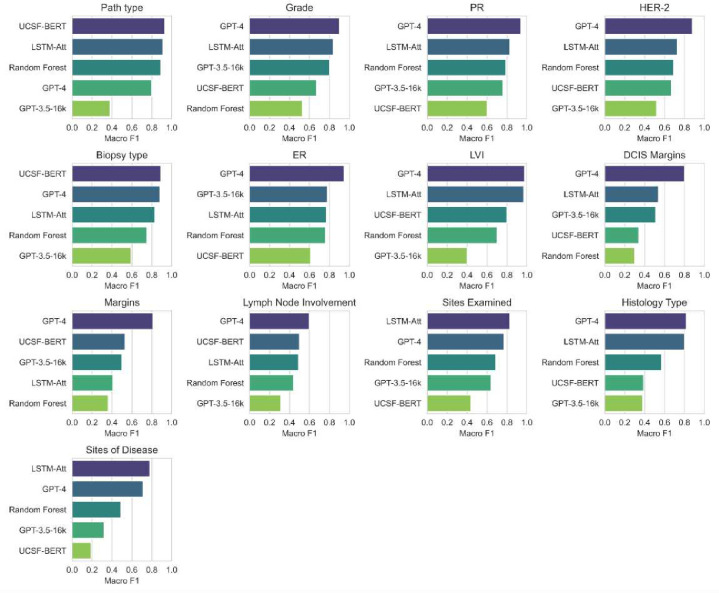
Classification performance, as measured by Macro F1, for different models for each classification task. All models other than GPT-3.5 and GPT-4 are trained in a supervised setup on task-specific training data. GPT-3.5 and GPT-4 models are evaluated zero-shot, i.e., in an unsupervised manner.

Despite no task-specific training, the GPT-4 model either outperformed or performed as well as our tasks-specific supervised models trained on task-specific breast cancer pathology data (Figure 4). For both the GPT-4 model and the GPT-3.5 model, all model responses could be automatically parsed as JSON without any errors. The average macro F1 score of the GPT-4 model across all tasks was 0.83, of the LSTM model with attention was 0.75, of the random forests model was 0.61, of the UCSF-BERT model was 0.58, and that of the GPT-3.5-turbo model (zero-shot) was 0.53. The GPT-4 model was significantly better than the LSTM model (the best supervised model) for the tasks of margins and estrogen receptor (ER) status classification (Approximate randomized testing for significance^27^, p < 0.01). These tasks encompass either a large training data imbalance resulting in a sparsity of class-specific training instances (margins) or could be frequently solved with an n-gram-matching approach (ER status). For all other tasks, no significant differences were obtained between the zero-shot GPT-4 model and the supervised LSTM model.

The GPT-3.5-turbo model performed significantly worse than the GPT-4 model for all tasks. Similarly, the UCSF-BERT model, which is a transformers model pre-trained on the UCSF notes corpus^22,23^, did not outperform simpler models like random forests or LSTM with attention for several tasks, potentially due to relatively small sample sizes and highly imbalanced training data. The random forests classifier performed well on keyword-oriented tasks, like pathology type classification and biomarker status classification, but under-performed on tasks requiring more advanced reasoning, like grade and margins inference.

### GPT-4 model error analysis

**Figure 5:**
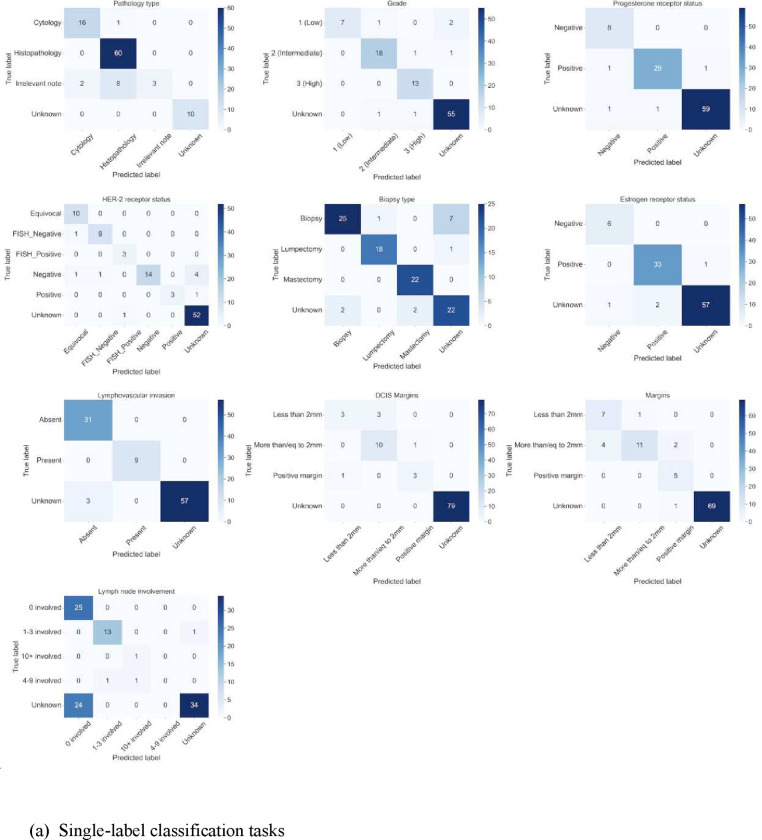

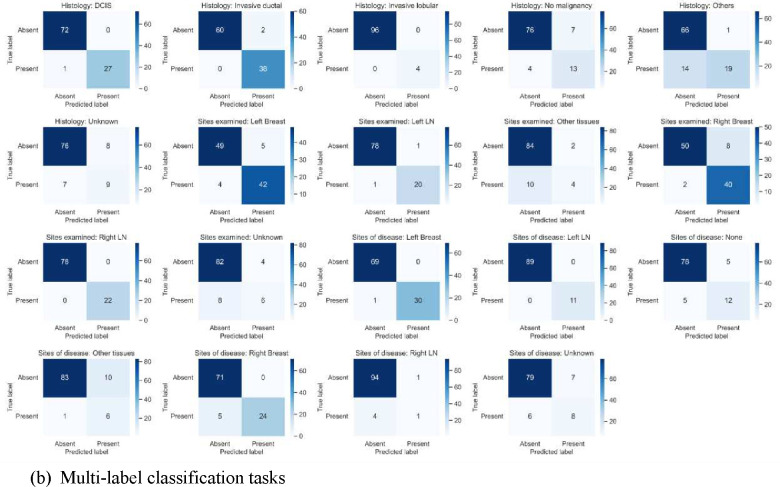
Confusion matrices for GPT-4 classification in (a) single-labeled tasks, (b) multi-labeled tasks.

As demonstrated in Figure 5, the confusion matrix of the GPT-4 model revealed that it had difficulties in differentiating the unknown class from the class that indicated no lymph node involvement and no lympho-vascular invasion. Furthermore, margins inference was complex for the model, where *more than 2mm margins* (negative margins) are confused with *less than 2mm* margins. Confusion between classes were more prevalent in multi-label tasks than single-label tasks. Further errors from the GPT-4 model were prevalent when the task design was ambiguous in model prompts, such as the grouping of sparse histology into an “others” category, the assignment of metastatic sites for breast cancer as “other tissues than breast or lymph nodes”, or the inference of pathology reports unrelated to breast cancer. The latter set of errors correspond to common sources of disagreements identified during the data annotation process.

Manual analysis of the GPT-4 model errors revealed several consistent sources of errors. Common sources of errors in biomarker reporting involved the reporting of results from clinical history or tests conducted at other sites that were not confirmed in the current report. Furthermore, the GPT-4 model incorrectly reported nuclear grade as the overall tumor grade when the overall grade was not discussed in the note. Moreover, common errors in reporting tumor margins were concerned with mathematical inferences over multiple margins (for example, anterior, posterior, medial, etc.), where the category representing the closest margin values needed to be provided. Manual analysis additionally uncovered several error sources for multi-label tasks. The model performed inconsistently when inferring sites of benign findings; while the model frequently missed reporting the site of benign findings as a site examined for tumors, it also sometimes included sites of benign findings as a site of cancer. Furthermore, sentinel and axillary lymph nodes were frequently reported as tissues other than breast or lymph nodes, although they were annotated as lymph node sites. Some errors related to complex cases were also found, for example, 1% staining results for progesterone receptors were provided as negative by the model, whereas they were annotated as positive. Finally, errors related to task setup were reflected in histology-related errors, where the model could not reliably abstain from providing histology from reports unrelated to breast cancer and from molecular pathology reports for ERBB2 despite being instructed as such, and errors due to the grouping of histologies like LCIS into an “others” category.

## Discussion

Task-specific supervised learning models trained on manually annotated data have been the standard approach in clinical NLP for over a decade^1^. Using a manually annotated dataset of 769 breast cancer pathology reports focused on the most clinically relevant report features, our study compared the performance of supervised learning models, including random forests classifier, LSTM models, and the UCSF-BERT model, with a zero-shot classification performance of two LLMs, the GPT-4 model, and the GPT-3.5-turbo model. We found that even in zero-shot setups, the GPT-4 model performs as well as or significantly better than simpler, task-specific supervised counterparts, although the GPT-3.5 model performs significantly worse than the GPT-4 model on all classification tasks. Previous studies have demonstrated similar results, showing that in zero-shot setups, LLMs consistently perform the same as or outperform fine-tuned models on biomedical NLP datasets with small training data sizes (fewer than 1000 training examples)^28,29^. Similar small datasets are common in medical informatics studies since domain expertise is frequently required for reliably annotating clinical notes, making the process time-consuming and difficult to scale^30^. This study enhances previous findings on a new real-world clinical dataset, reinforcing that LLMs are promising for use in classification tasks in low-resource clinical settings.

Tasks where the training data contained high class imbalance or keyword-based tasks (i.e., could be largely solved with simple lexical matching) were particularly conducive for using GPT-4 model over task-specific supervised models. Given that the GPT-4 model is already trained on internet-scale corpora, the model may already encode a fundamental understanding of breast cancer pathology-related terminology, which may explain its surprising zero-shot capability on these tasks, including that on complex and imbalanced tasks like margins inference. However, the reasons behind the striking performance difference between the GPT-3.5 and GPT-4 models remain unclear due to the closed nature of these models, although similar trends have been observed in previous medical NLP studies^31,32,13^.

An analysis of the GPT-4 model errors indicated several errors due to insufficient understanding of idiosyncratic task-design choices, for example differentiating between “Unknown” and “no lymph node involvement” categories, and grouping of less frequent histologies into an “others” category. It is possible that these errors can be mitigated with strategies, such as few-shot learning to demonstrate a better understanding of annotation-specific choices, or chain-of-thought-prompting to elucidate reasoning and avoid answering from incomplete or old information within text report. However, it has been demonstrated earlier that the GPT-4 model cannot process long input contexts efficiently, particularly when the results are included in the central part of the context^33^. Hence, how to best integrate in-context learning with long pathological notes such that the model can still make effective use of the supported context length remains to be investigated in future research.

Although this study compared two proprietary LLMs with supervised classifiers on a real-world breast cancer dataset, several design choices may have impacted the findings. The dataset was curated from a single health system, and further validation of the findings on pathology reports from other health systems may improve the reliability of the results. Although potential de-identification errors may have impacted the capability of LLMs, the data reflects real-world setups for retrospective observational studies in a privacy-preserving manner. Furthermore, although some model-specific hyperparameters were explored for baseline models, all possible choices have not been explored, and it may be possible to improve baseline model performance further with continued development. Moreover, LLMs used in the study were evaluated using a single prompt and model setting, and the results could be sensitive to these design choices. However, the findings of this study will inform future studies on the development of more advanced prompting and few-shot strategies for LLMs to obtain even better performance, the development of effective annotated datasets for simpler supervised classification setups, the evaluation of newer LLMs for clinical information extraction, and the analysis of output sensitivity to input prompts and model settings. While the findings from this study demonstrate the promising capability of LLMs for clinical research, if access to models like the GPT-4 model is prohibitive due to either privacy or computational constraints, comparable performance on EHR-based NLP tasks such as pathology classification can be obtained with simpler deep learning classifiers, particularly if annotated sample sizes are sufficiently large and class imbalance can be controlled through targeted annotations of minority classes for model training. Finally, the studied classifiers may exhibit biases against specific demographics, and caution must be exercised when deploying them in clinical workflows. These biases need to be investigated further in the future to establish concrete guidelines for their use.

Despite widespread studies in oncology information extraction from textual clinical records^34,35^, annotated datasets of breast cancer pathology reports are not publicly available. To make the findings of this study replicable and promote further research on breast cancer pathology extraction, the dataset curated in this study along with corresponding source code for the supervised machine learning pipelines and zero-shot LLM inference will be shared publicly through a controlled-access repository PhysioNet, accessible via a data use agreement. Providing this large, annotated dataset for future research increases the impact of this work by allowing it to serve as a baseline of comparison as LLMs continue their rapid progress.

## Conclusions

The study compared breast cancer pathology classification abilities of five models of varying sizes and architecture, finding that the GPT-4 model, even in zero-shot setups, performed similarly to or better than the LSTM model with attention trained on nearly 550 pathology report examples. The GPT-4 model outperformed simpler baselines for classification tasks with high class imbalance or that required simpler keyword-matching for inference. However, when large training datasets were available, no significant difference was observed between the performance of simpler models like the LSTM model with attention compared to the GPT-4 model. The results of this study demonstrated that while LLMs may relieve the need for resource-intensive data annotations for creating large training datasets in medicine, if there are privacy, computational, or cost-related concerns regarding the use of LLMs with patient data, it may be possible to obtain reliable performance with simpler models by developing large annotated datasets, with particular focus on minority class labeling potentially in an active-learning setup.

## Supplementary Material

Supplement 1

## Figures and Tables

**Table 1.** Socio-demographic distribution of patients in the annotated dataset

Sample characteristic	Count (percentage) (n=769)	

Gender		
Male	7	(0.91%)
Female	762	(99.09%)
Age		
Median [IQR]	55.0	[19.0]
Race/ethnicity		
White	505	(65.67%)
Asian	101	(13.13%)
Latinx	42	(5.46%)
Black or African-American	36	(4.68%)
Native Hawaiian or Other Pacific Islander	7	(0.91%)
Southwest Asian and North African	2	(0.26%)
Other	23	(2.99%)
Multi-Race/Ethnicity	15	(1.95%)
Unknown/Declined	38	(4.94%)
Language		
English	702	(91.29%)
Russian	18	(2.34%)
Unknown/Declined	17	(2.21%)
Chinese - Cantonese	9	(1.17%)
Spanish	9	(1.17%)
Vietnamese	4	(0.52%)
Chinese - Mandarin	2	(0.26%)
Burmese	1	(0.13%)
Italian	1	(0.13%)
Cambodian	1	(0.13%)
Samoan	1	(0.13%)
Korean	1	(0.13%)
Farsi	1	(0.13%)
Sign Language	1	(0.13%)
Other	1	(0.13%)

Abbreviation: IQR, Inter-quartile range

**Table 2:** Inter-annotator agreement between expert clinical annotators, as quantified by Krippendorf’s alpha score for single-label and multilabel tasks.

Task	Inter-annotator agreement (Krippendorf’s alpha)

Biopsy type	0.80
Num lymph nodes involved	0.89
ER	0.85
PR	0.90
HER2	0.80
Grade	0.85
LVI	0.97
Margins	0.93
DCIS Margins	0.77
Histology (Multilabel)	0.82
Sites examined (Multilabel)	0.79
Sites of disease (Multilabel)	0.85

AVERAGE	**0.85**

## Footnotes

1 The AI framework to safely use OpenAI application programming interfaces is called Versa at UCSF.

2
https://physionet.org/news/post/415

3 Krippendorf’s alpha was preferred over Cohen’s kappa to quantify inter-annotator agreement on multi-label annotation tasks in addition to single-label tasks.

## References

[1] Wu H. A survey on clinical natural language processing in the United Kingdom from 2007 to 2022. npj Digit. Med. 5, 1–15 (2022). 36544046 10.1038/s41746-022-00730-6 PMC9770568

[2] Fu S. Recommended practices and ethical considerations for natural language processing-assisted observational research: A scoping review. Clin Transl Sci 16, 398–411 (2023). 36478394 10.1111/cts.13463 PMC10014687

[3] Brown T. Language Models are Few-Shot Learners. in Advances in Neural Information Processing Systems vol. 33 1877–1901 (Curran Associates, Inc., 2020).

[4] Kojima T., Gu S. (Shane), Reid M., Matsuo Y. & Iwasawa Y. Large Language Models are Zero-Shot Reasoners. Advances in Neural Information Processing Systems 35, 22199–22213 (2022).

[5] Agrawal M., Hegselmann S., Lang H., Kim Y. & Sontag D. Large language models are few-shot clinical information extractors. in Proceedings of the 2022 Conference on Empirical Methods in Natural Language Processing 1998–2022 (Association for Computational Linguistics, Abu Dhabi, United Arab Emirates, 2022).

[6] Eriksen A. V., Möller S. & Ryg J. Use of GPT-4 to Diagnose Complex Clinical Cases. NEJM AI 1, AIp2300031 (2023).

[7] Wang Z. Can LLMs like GPT-4 outperform traditional AI tools in dementia diagnosis? Maybe, but not today. Preprint at http://arxiv.org/abs/2306.01499 (2023).

[8] Barile J. Diagnostic Accuracy of a Large Language Model in Pediatric Case Studies. JAMA Pediatrics (2024) doi:10.1001/jamapediatrics.2023.5750. PMC10762631 38165685

[9] Liu Q. Exploring the Boundaries of GPT-4 in Radiology. in Proceedings of the 2023 Conference on Empirical Methods in Natural Language Processing (eds. Bouamor H., Pino J. & Bali K.) 14414–14445 (Association for Computational Linguistics, Singapore, 2023). doi:10.18653/v1/2023.emnlp-main.891.

[10] Fink M. A. Potential of ChatGPT and GPT-4 for Data Mining of Free-Text CT Reports on Lung Cancer. Radiology 308, e231362 (2023). 37724963 10.1148/radiol.231362

[11] Alsentzer E. Zero-shot interpretable phenotyping of postpartum hemorrhage using large language models. npj Digit. Med. 6, 1–10 (2023). 38036723 10.1038/s41746-023-00957-x PMC10689487

[12] Guevara M. Large language models to identify social determinants of health in electronic health records. npj Digit. Med. 7, 1–14 (2024). 38200151 10.1038/s41746-023-00970-0 PMC10781957

[13] Sushil M. CORAL: Expert-Curated medical Oncology Reports to Advance Language Model Inference. Preprint at 10.48550/arXiv.2308.03853 (2024). PMC12007910 40255242

[14] Wong C. Scaling Clinical Trial Matching Using Large Language Models: A Case Study in Oncology. Preprint at 10.48550/arXiv.2308.02180 (2023).

[15] Datta S. AutoCriteria: a generalizable clinical trial eligibility criteria extraction system powered by large language models. Journal of the American Medical Informatics Association 31, 375–385 (2024). 37952206 10.1093/jamia/ocad218 PMC10797270

[16] Mirza F. N. Using ChatGPT to Facilitate Truly Informed Medical Consent. NEJM AI 0, AIcs2300145 (2024).

[17] Radhakrishnan L. A certified de-identification system for all clinical text documents for information extraction at scale. JAMIA Open 6, ooad045 (2023). 37416449 10.1093/jamiaopen/ooad045 PMC10320112

[18] OpenAI. GPT-4 Technical Report. Preprint at 10.48550/arXiv.2303.08774 (2023).

[19] Breiman L. Random Forests. Machine Learning 45, 5–32 (2001).

[20] Hochreiter S. & Schmidhuber J. Long Short-Term Memory. Neural Comput. 9, 1735–1780 (1997). 9377276 10.1162/neco.1997.9.8.1735

[21] Bahdanau D., Cho K. & Bengio Y. Neural Machine Translation by Jointly Learning to Align and Translate. Preprint at 10.48550/arXiv.1409.0473 (2016).

[22] Sushil M., Ludwig D., Butte A. J. & Rudrapatna V. A. Developing a general-purpose clinical language inference model from a large corpus of clinical notes. Preprint at http://arxiv.org/abs/2210.06566 (2022).

[23] Silverman A. L. Algorithmic identification of treatment-emergent adverse events from clinical notes using large language models: a pilot study in inflammatory bowel disease. 2023.09.06.23295149 Preprint at 10.1101/2023.09.06.23295149 (2023). PMC11090709 38459719

[24] Bojanowski P., Grave E., Joulin A. & Mikolov T. Enriching Word Vectors with Subword Information. Transactions of the Association for Computational Linguistics 5, 135–146 (2017).

[25] Ridnik T. Asymmetric Loss For Multi-Label Classification. in 2021 IEEE/CVF International Conference on Computer Vision (ICCV) 82–91 (IEEE, Montreal, QC, Canada, 2021). doi:10.1109/ICCV48922.2021.00015.

[26] Krippendorff K. Content Analysis: An Introduction to Its Methodology. (SAGE Publications, 2018).

[27] Edgington E. S. Approximate Randomization Tests. The Journal of Psychology 72, 143–149 (1969).

[28] Jahan I., Laskar M. T. R., Peng C. & Huang J. A Comprehensive Evaluation of Large Language Models on Benchmark Biomedical Text Processing Tasks. Preprint at http://arxiv.org/abs/2310.04270 (2023). 10.1016/j.compbiomed.2024.108189 38447502

[29] Chen Q. Large language models in biomedical natural language processing: benchmarks, baselines, and recommendations. Preprint at 10.48550/arXiv.2305.16326 (2024).

[30] Gao Y. A scoping review of publicly available language tasks in clinical natural language processing. Journal of the American Medical Informatics Association 29, 1797–1806 (2022). 35923088 10.1093/jamia/ocac127 PMC9471718

[31] Taloni A. Comparative performance of humans versus GPT-4.0 and GPT-3.5 in the self-assessment program of American Academy of Ophthalmology. Sci Rep 13, 18562 (2023). 37899405 10.1038/s41598-023-45837-2 PMC10613606

[32] Nori H. Can Generalist Foundation Models Outcompete Special-Purpose Tuning? Case Study in Medicine. Preprint at http://arxiv.org/abs/2311.16452 (2023).

[33] Liu N. F. Lost in the Middle: How Language Models Use Long Contexts. Preprint at 10.48550/arXiv.2307.03172 (2023).

[34] Wang L. Assessment of Electronic Health Record for Cancer Research and Patient Care Through a Scoping Review of Cancer Natural Language Processing. JCO Clin Cancer Inform e2200006 (2022) doi:10.1200/CCI.22.00006. 35917480 PMC9470142

[35] Gholipour M., Khajouei R., Amiri P., Hajesmaeel Gohari S. & Ahmadian L. Extracting cancer concepts from clinical notes using natural language processing: a systematic review. BMC Bioinformatics 24, 405 (2023). 37898795 10.1186/s12859-023-05480-0 PMC10613366

